# Assessment of DSMs Using Backpack-Mounted Systems and Drone Techniques to Characterise Ancient Underground Cellars in the Duero Basin (Spain)

**DOI:** 10.3390/s19245352

**Published:** 2019-12-04

**Authors:** Serafín López-Cuervo Medina, Enrique Pérez-Martín, Tomás R. Herrero Tejedor, Juan F. Prieto, Jesús Velasco, Miguel Ángel Conejo Martín, Alejandra Ezquerra-Canalejo, Julián Aguirre de Mata

**Affiliations:** 1 Departamento de Ingeniería Topográfica y Cartográfica, Escuela Técnica Superior de Ingenieros en Topografía, Geodesia y Cartografía, Universidad Politécnica de Madrid, Campus Sur, A-3, Km 7, 28031 Madrid, Spain; juanf.prieto@upm.es (J.F.P.); jesus.velasco@upm.es (J.V.); julian.aguirre@upm.es (J.A.d.M.); 2 Departamento de Ingeniería Agroforestal, Escuela Técnica Superior de Ingeniería Agronómica, Alimentaria y de Biosistemas, Universidad Politécnica de Madrid, Av. Puerta de Hierro, 2, 28040 Madrid, Spain; enrique.perez@upm.es (E.P.-M.); tomas.herrero.tejedor@upm.es (T.R.H.T.); miguelangel.conejo@upm.es (M.Á.C.M.); 3 Departamento de Ingeniería y Gestión Forestal y Ambiental, Escuela Técnica Superior de Ingeniería de Montes y del Medio Natural, Universidad Politécnica de Madrid, Ciudad Universitaria, 28040 Madrid, Spain; alejandra.ezquerra@upm.es

**Keywords:** DSM assessment, backpack mobile mapping, UAV, underground cellars

## Abstract

In this study, a backpack-mounted 3D mobile scanning system and a fixed-wing drone (UAV) have been used to register terrain data on the same space. The study area is part of the ancient underground cellars in the Duero Basin. The aim of this work is to characterise the state of the roofs of these wine cellars by obtaining digital surface models (DSM) using the previously mentioned systems to detect any possible cases of collapse, using four geomatic products obtained with these systems. The results obtained from the process offer sufficient quality to generate valid DSMs in the study area or in a similar area. One limitation of the DSMs generated by backpack MMS is that the outcome depends on the distance of the points to the axis of the track and on the irregularities in the terrain. Specific parameters have been studied, such as the measuring distance from the scanning point in the laser scanner, the angle of incidence with regard to the ground, the surface vegetation, and any irregularities in the terrain. The registration speed and the high definition of the terrain offered by these systems produce a model that can be used to select the correct conservation priorities for this unique space.

## 1. Introduction

Instruments and techniques for the massive capture of data are increasingly being used to document all types of cultural landscapes and heritages. There are recommendations and criteria for adequate procedures to ensure the defence and preservation of these types of heritages and landscapes in the European context [1]. Many research projects on heritage management use geospatial technologies [2,3,4,5] to generate various products such as digital surface models (DSM) [6,7,8,9,10,11,12,13]. A large number of studies focus on optimising and improving actions with unmanned aircraft vehicles (UAV) [14].

**Figure 1:**
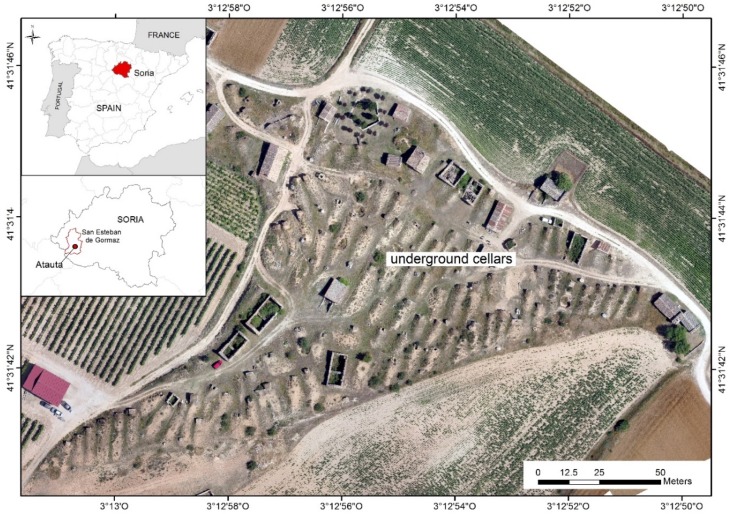
Location of the study area. The underground cellars are located in Atauta (Soria), a region in the Duero Basin in the north-central part of Spain.

Against this background, our study zone, the underground cellars of El Plantío in Atauta (Soria), declared an Asset of Cultural Interest (Bien de Interés Cultural, BIC) in March 2017, which represents a unique testimony of the life associated with work on the land and the traditional wine production system. It is important to ensure that they are not ultimately forgotten and destroyed, as they are a manifestation of the cultural identity of a large region in the Duero River basin (Figure 1).

In recent years, fixed terrestrial laser scanning (TLS) systems have been used to map and monitor various areas of interest from the point of view of their cultural heritage [15,16,17,18], in archaeological studies [19,20], underground studies [21,22], and civil engineering [23], among others [24]. 3D technologies include both the TLS system and sensors installed on UAV for the documentation, visualisation, and preservation of heritage [25,26,27]. The precision of a digital surface model (DSM) obtained by UAV photogrammetry for documenting surface structures [28] and forms in 3D models [29,30,31] and for identifying constructions in urban planning [32,33] has also been analysed. Chiabrando et al. [19] described a technique for preparing digital surface models in their archaeological studies. Norhafizi validated the use of UAV for creating DSMs of tide data [34]. Villanueva [35] studied DSMs and their application in zones at risk of flooding.

Komarek et al. [36] carried out studies to assess the precision of DSMs obtained by UAV on rural plots. In other cases, DSMs have been assessed with or without ground control points (GCP) taken by means of a real-time kinematic Global Positioning System (RTK-GPS) techniques [37,38,39]. Other studies have evaluated the use of software for generating DSM models from UAV with GCP taken in the field by means of other geomatic methods such as TLS, GPS [40], or UAV photogrammetry techniques [41].

There are examples of studies on the precision of 3D models in indoor spaces and areas through backpack mobile mapping [42,43,44] and assessments of different TLS [45,46,47]. Other works assess the performance of a mobile mapping system (MMS) and backpack mobile mapping [48,49] compared to the use of a TLS [47], and highlight the superior performance of the mobile system, even though its overall precision is lower than with TLS. Campos [50] applied the backpack mobile mapping system in forest mapping where there is limited accessibility. Przemyslaw [51] also used backpack mobile mapping for the geolocation of tree trunks and their comparison with UAV records.

The DSMs obtained are used to compare and complement the information registered with ground penetrating geo-radar technology (GPR) and terrestrial laser scanning (TLS) [52]. In previous studies in the same area [53], DSMs enabled the definition and comparison of wall and ceiling thicknesses in underground cellars to ensure their stability.

The aim of this work is to assess the condition of the roofs of underground cellars in their natural state by obtaining an accurate DSM to characterise, detect, and prevent their collapse. This is accomplished using a backpack-mounted 3D mobile scanning system (backpack MMS) and a fixed-wing drone (UAV).

The study examines the use of backpack MMS in areas of irregular terrain such as the Atauta underground cellar, using data registration parameters and DSM generation including working widths and angles of incidence in the measurement and obstacles. The study also compared it with UAV equipment that enables analysis of the benefits of both systems for generating DSMs of key importance in assessing the current risk status of underground wine cellars.

## 2. Materials and Methods

### 2.1. Case Study Description

The study area is part of the series of underground wine cellars of El Plantío in Atauta, Soria (41°31′ N, 3°12′ W), shown in Figure 1, which were declared an Asset of Cultural Interest (BIC) as an “ethnographic collection” on 16 March 2017. Located at the foot of the village of Atauta on an area of 1.9 ha, they are a testimony of the life associated with work on the land and the artisanal wine production system. These underground constructions were used to store and preserve wine.

### 2.2. Equipment Used to Take the Images

**Figure 2:**
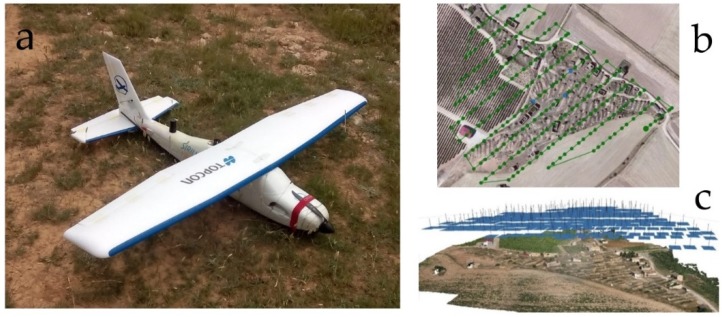
(**a**) Mavinci UAV used during the registration process. (**b**) Image orientation process task in Agisoft Photoscan^®^ professional software (http://www.agisoft.com/) showing flight paths and photocenters. (**c**) 3D view diagram of the recorded images.

The topographic and photogrammetric survey using UAV techniques was carried out with a Mavinci fixed-wing drone with a Lumix-GX1 camera with a focal distance of 14 mm and a resolution of 4592 × 3448 pixels. The flight was made at a height of 90 m, obtaining a ground sample distance (GSD) of 2.16 cm and taking 344 images by following a flight plan with a regular grid pattern defined in a northwest-southeast direction. The longitudinal overlap was 70%, and the transversal overlap was 50%. Precise coordinates were obtained throughout the flight due to an RTK-GPS receiver located on the UAV, which received corrections from a base on the ground. Also using RTK-GPS techniques, high-precision control points were observed in order to improve the geo-referencing of the photogrammetric model with points on the ground and for a quality check assessment. The deformation in the images caused by the camera [54] is compensated with an autocalibration performed during the aerotriangulation process (Figure 2).

### 2.3. System Used for Mass Registration with a Laser

**Figure 3:**
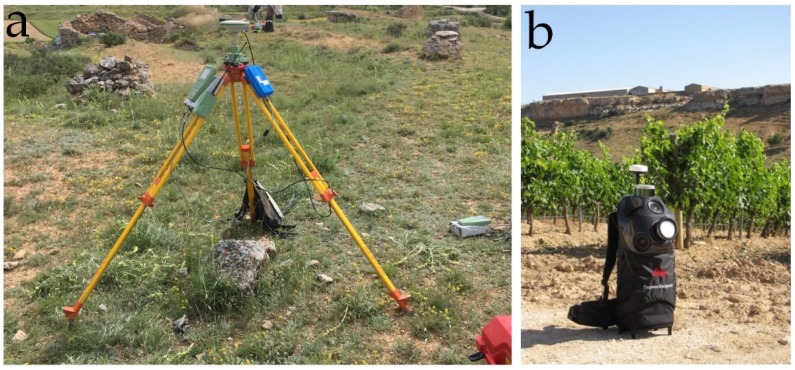
(**a**) GPS Leica GX1230 GG equipment, acting as a reference base. (**b**) Pegasus backpack system for taking the point cloud in the study area before obtaining the DSM model to be assessed.

A Leica Pegasus data registration system was used as a backpack mobile mapping model (Figure 3). This system incorporates five cameras and two LiDAR (light detection and ranging) scanners for the registration of the 3D point cloud and images. It also has two Velodyne sensors (VLP16) that rotate at 10 Hz and acquire 600,000 points per second at a distance of up to 100 m, even though this may be influenced by several factors, as indicated in its specifications [48,55]. It weighs approximately 13 kg and has a scanning autonomy of three hours. The system includes a SLAM (simultaneous localisation and mapping) algorithm and an IMU (inertial measurement unit) as an aid for generating the 3D model.

Leica Pegasus Backpack allows the acquisition of LiDAR and image data with precise positioning of outdoor and indoor data [18] in a system that is easily transportable by one person. This makes this type of equipment very useful in environments with limited space, underground environments, [20] and in areas with dense vegetation, as well as for managing data on disasters [56] and documenting industrial facilities. It offers the option of including external sensors such as GPR equipment, thermal cameras, noise and pollution sensor, etc., assisted by a flash module for working in conditions of low light. The work software has tools for extracting LiDAR and photogrammetric data and detecting changes, and is compatible with workflows by means of AutoCAD and ArcGIS. The only limitation to its use is its autonomy of four hours due to its batteries.

It took 30 min to configure and calibrate the backpack MMS data registration system, and one hour to survey the study area. The main features of the UAV and the Pegasus backpack are shown in Table 1.

### 2.4. Control and Assessment System Obtained by GPS

**Figure 4:**
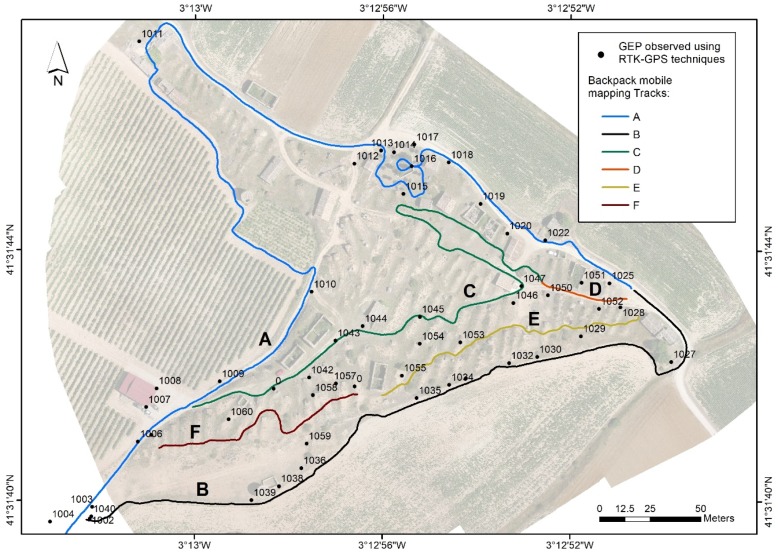
GEP observed using RTK-GPS techniques for the characterisation surveys of collapse zones. Lines depict the backpack mobile mapping tracks (Tracks A to F) used to assess the 3D model.

High-precision coordinates were computed on a fixed base using GPS techniques in post-processing to obtain both Ground Evaluation Points (GEP) and Ground Control Points (GCP). The points are distributed to cover the entire area of characterisation and possible collapses on the surface of the underground cellars, which are the same as the UAV flight and the tracks taken by the backpack, as shown in Figure 4.

**Figure 5:**
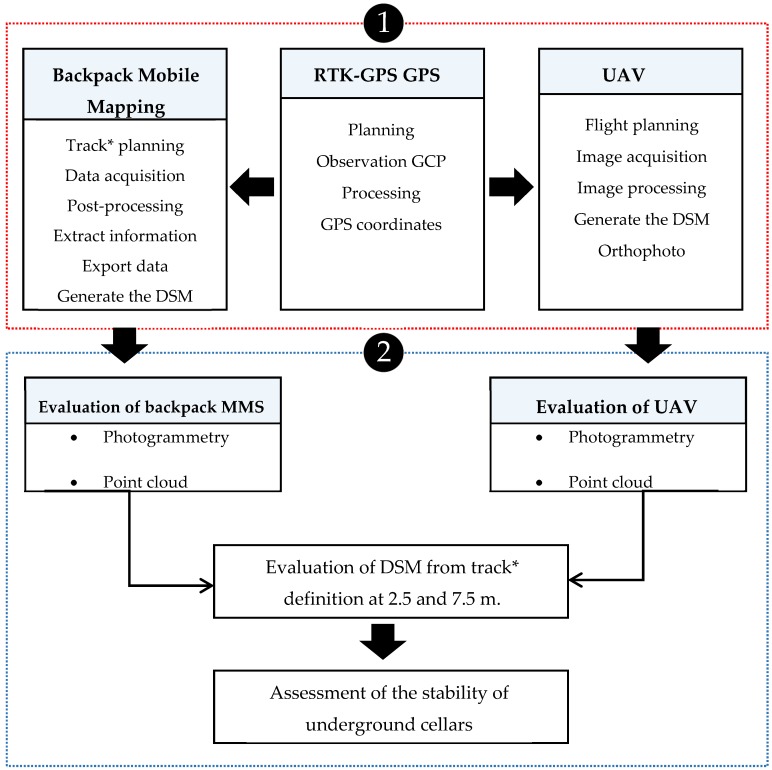
Workflow of the methodology for data acquisition where (**1**) refers to data acquisition and post-processing for backpack mobile mapping, UAV, and RTK-GPS, and (**2**) represents DSM processing and stability evaluation. Note the interactions between the benchmark survey (GPS) and backpack mobile mapping and UAV surveys.

This GPS base served as a reference for all the topographic and photogrammetric surveys (see Figure 5, Section 1, centre). The equipment used was a Leica Geosystems GX1230 GG, and the data were processed with the Leica Geo Office software, with a relative average precision of more than 0.02 m in all the points. The base GPS receiver was situated in the centre of the study area and all the GCPs and GEPs observed were located at a radius of less than 200 m and measured with another two GPS rover receivers. From this base, 12 GCPs were computed in post-processing to support the UAV. The GCPs were positioned on the periphery and inside the study zone. This same base served as a support for the backpack MMS, and all its tracks were within a range of less than 250 m from the base.

Furthermore, 59 GEP coordinates were also computed by RTK-GPS techniques from the same base, and, subsequently, used in the assessment of the DSMs generated.

### 2.5. Methods

As stated, the aim of this research is to obtain digital surface models (DSM) on the terrain corresponding to the natural cover of the underground cellars. A methodology was defined in several phases for this purpose, as shown in the diagram in Figure 5. In the first phase, the 45 GEP points were analysed after obtaining the data with methods based on backpack MMS and UAV technologies. These points were identified using Agisoft Photoscan^®^ Professional software and included in the aerotriangulation process, and then identified and extracted from the DSM generated from the dense point cloud in the UAV photogrammetric project. Each GEP point was located in the data from the photographs registered by backpack MMS and measured by stereoscopy with ArcExplorer (Esri, USA) software. The GEP were also identified in the LiDAR point cloud from the backpack MMS. These four measures were compared with the GEP coordinates obtained by the RTK-GPS method. 

The results of the comparisons led to the selection of the point cloud-based methods and the rejection of the photogrammetric methods, since they were insufficiently dense to generate DSMs that could be guaranteed to detect possible collapses. However, the photogrammetric backpack MMS was the most accurate method (see results section). 

The data obtained with the backpack MMS define the tracks from which to obtain the point cloud covering the terrain. These tracks were used as a common element to establish the zones that cover the total study area and allow the evaluation of the DSM.

In a second phase, the results from the two massive data recording techniques are statistically analysed to compare the numerical and graphic products obtained and to define the DSM more clearly.

As has been mentioned, a supported network of control points was defined with RTK-GPS to serve as the basis for the UAV flight and the MMS backpack. This network established the real precision of both models and allowed the study of parameters such as the distance to the track in the case of backpack MMS, the point density according to the method used, and the veracity of the model with regard to walls, roofs, steeply sloping areas, and other elements. These points acted as a geometric control of the parameters to be assessed. The methodology applied in each system is shown in the diagram in Figure 5 and described below in more detail.

### 2.6. Processing the UAV Point Cloud

The previously mentioned software was used to generate the point cloud with the images recorded with UAV, and also included their corresponding metadata and the RTK-GPS coordinates of the photocentres of the images referenced on the ground. This type of software uses sfM-MVS algorithms for the orientation and calculation of point clouds and is widely used in UAV work processes due to the large number of images, type of cameras, and auxiliary data taken into account [57,58,59,60].

The sfM-MVS processing workflow used was as follows: initially a feature detection is performed by identifying a large number of key points in each image. With these points, an image matching process is carried out to identify and match these features in all the images in which they are registered. Subsequently, a blunder adjustment is performed with the camera’s self-calibration, photocenter coordinates, and other parameters. Thus, the photogrammetric solution of the external orientation parameters of the images is obtained together with the 3D sparse model composed of the feature points detected in these images, as described in Reference [61].

This study has specifically included the use of GCPs as field control, GEPs as manual photogrammetric tie points, and feature points as automatic tie points or sparse points (see Figure 5, right) in the same photogrammetric adjustment process. Lastly, the digital terrain model (DTM) was created to produce the orthophotograph on which to identify the assessment points and verify the quality of the LiDAR DSM.

### 2.7. Processing the Backpack MMS Point Cloud

The point clouds from the LiDAR data registered with the backpack MMS platform were computed using the Leica Pegasus Manager software, which also allows the management of the data captured by means of MMS. It is composed of several modules in a workflow that ranges from the prior planning of the work to be done, the acquisition of the data, the subsequent processing and refinement with other sensors and algorithms, and the automatic and manual extraction of the characteristics of interest within the point clouds.

A total of six tracks were obtained (labelled A to F, Figure 4). The points were measured by photogrammetry with the Leica ArcExplorer application. The software includes a tool that allows the stereoscopic measurement of any point in the images from the perspective centres recorded and their rotations, and, thereby, obtains the coordinates of the GEPs. The last step was to identify the GEPs on the orthophotograph obtained by UAV and to measure them in the LiDAR MMS point cloud (see Figure 5, left).

### 2.8. Comparison of the UAV-Backpack MMS Point Clouds

After comparing the coordinates for each GEP with the DSMs obtained from the UAV and backpack MMS data, the information from each registration system was analysed. This comparison uses the tracks defined with backpack MMS to identify the different sections for analysis, while taking into account the following factors.

- The surface of the terrain in its natural state is covered with herbaceous and shrubby vegetation, which may pose an obstacle for measurement in both methods. Two types of zone were identified to assess the DSMs: one with exterior corridors with less influence of vegetation, and another on the interior with tracks with a greater presence of grassland vegetation. Some tracks were made to pass over the roofs of the cellars in order to obtain more detailed information in critical areas.
- Taking the axis of the tracks as a reference for the LiDAR scanning distances, the point cloud obtained on both sides of these tracks was computed and analysed to a limit of between 2.5 and 7.5 m from their axis.

The backpack MMS tracks A-F were used as identifiers for the assessment, and grouped according to the similarity of their features. Tracks A and B are perimetral, defined in areas of broad corridors with little influence of vegetation at a distance of 2.5 m each side of the axis. Tracks C, D, E, and F are located on the interior and, therefore, have a presence of vegetation and irregularities in the terrain, low walls, etc. These six tracks were divided into sections based on the similarity of the type of terrain in order to enable a better comparison between UAV and MMS data. Hence, Track A was divided into five sections, Track B was divided into four sections, Tracks C and E were divided into two sections, and, lastly, Tracks D and F each have only one section. Obstacles caused by constructions were eliminated during the digital processing of the point cloud to avoid errors due to vertical and/or horizontal measuring in the two systems.

For the assessment, a DSM was generated with a resolution of 0.05 m from the point clouds obtained with backpack MMS, and a grid with a point-to-point resolution of 0.10 m was projected on the DSM. The same procedure was followed for the UAV point cloud. Therefore, the resolution and geolocation of the DSMs coincide and allow their subsequent comparison.

Lastly, for the assessment of the DSM comparison at 7.5 m from the axis of the track taken by backpack MMS, the sections with no errors due to obstacles were maintained, and sections A4, A5, B1, B2, and C2 were removed.

## 3. Results

Before comparing the results of the different methods, it should be noted that a mean square error of 1.9 cm in height was obtained in the calculation of the GEP points.

Ten of the 59 initial GEPs were eliminated since they could not be measured in all four methods including some that were impossible to identify and others had insufficient resolution for providing real coordinates with regard to other methods, such as the eaves of a warehouse in the testing area. Four more points were also eliminated due to problems in identifying them, such as the corner of a bench which—although it could be adequately measured in photogrammetry—was difficult to discern from the LiDAR point clouds.

The following results were obtained from the various methods, according to the distance criteria 0 to 2 m, 2 to 3.5 m, and 3.5 to 10 m.

### 3.1. Point Cloud Processed with UAV

**Figure 6:**
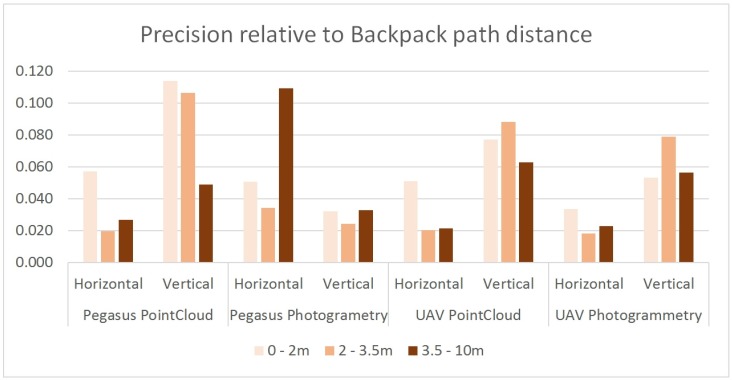
Comparison of precision based on the method used and the distance from the axis of backpack MMS.

- The assessment of coordinates was obtained by triangulation with the UAV images. The GEP points included as linkage points in the aerotriangulation process gave the following results. The mean square error for the three distances is 4.7 cm in distance and 8.9 cm in height. The points were measured on an average of 16 photographs and their internal precision was 1.2 pixels. The standard deviation was up to 6.2 cm in height. This is a “low density” point cloud (2 points/square metre) (Table 2 and Figure 6). Five minutes were required to measure each point in photogrammetry, since the points must be measured in all the photographs. Sixty points were recorded.
- This section discusses the assessment of points in the UAV dense point cloud. The GEPs identified and measured in the dense point cloud gave the following results. The mean square error for the three distances was 5.0 cm in distance and 10.7 cm in height. The standard deviation of the points measured in the dense point cloud was 7.5 cm (Table 2). The points in the dense clouds were measured. Ten hours were required to make the dense model of 2,400,000 points extracted from this work.

### 3.2. Point Cloud Processed with Backpack MMS

- Assessment of LiDAR points recorded by backpack MMS. The mean square error for the three distances was 5.2 cm in distance and 7.0 cm in height. The standard deviation in height was up to 9.2 cm (Table 2 and Figure 6). The point measurement with MMS is an automatic and dynamic process, and the points are obtained while covering the work zone. Two hours were required to record the 115 million points included in the trajectories in the project.
- The assessment of coordinates was obtained by photogrammetry with ArcExplorer software. The mean square error for the three distances was 10.3 cm in distance and 3.6 cm in height. The standard deviation in height was up to 3.2 cm (Table 2). This is the least productive measuring process, with one point measured every 10 min. The points are identified manually, and the parallax must be manually cancelled in each point.

Table 2 shows the precision and densities obtained with each method. They are then compared with the RTK-GPS points network measured in the area, and the data are grouped by distance to the track with the laser scanner located in the backpack: 0 to 2 m (measurement angle of the laser scanner on the terrain from 90° to 5°), 2 to 3.5 m (measurement angle of 45° to 22°) and over 3.5 m (angle of less than 22°). The greatest precisions, particularly in terms of height, are obtained in the photogrammetric measurement on the backpack images, even though this is the least efficient method in terms of density and production. When the point is clearly defined, no significant loss of precision was observed with regard to distance.

### 3.3. Comparison of Backpack MMS vs. UAV DSMs

**Figure 7:**
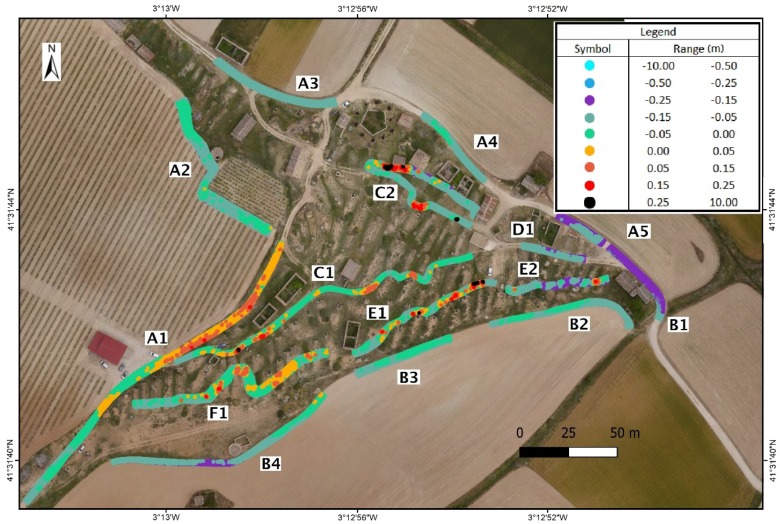
Distribution of the precise comparison of the point clouds based on the backpack MMS track. Letters denote the different tracks while numbers depict the different sections in the tracks.

The result of the precision analysis for the DSM at a distance of 2.5 m from the axis of the track is shown in Table 3 and Figure 7. The two DSMs are evaluated with a square grid with a resolution of 0.1 m based on the point clouds obtained with UAV and backpack MMS. The height precision for the tracks on the exterior is 3.8 cm, whereas the precision in interior areas is 5.7 cm in height. Tracks A4, A5, B1, B4, D1, and E2 have a vertical displacement between 0.10 and 0.15 m. All these areas have an influence due to construction walls or other obstacles.

**Figure 8:**
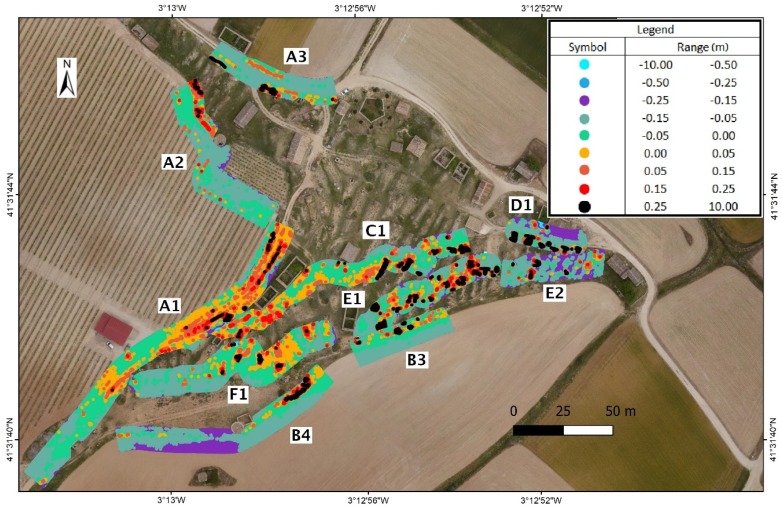
Precision distribution with the influence of 7.5 m with regard to the backpack MMS track. Letters denote the different tracks while numbers depict the different sections in the tracks.

If the distance from the backpack MMS track increases from 2.5 m to 7.5 m on each side of the axis from which the point clouds defining the DSM are obtained, the maximum angle of measurement of backpack MMS on the terrain would decline from 45° to 20°, which increases the error due to the noise produced in the measurement. There are also a higher number of errors due to obstacles. The number of zones in the study was, therefore, reduced in order to avoid errors caused by built elements or obstacles. The study for these distances at 7.5 m is limited to zones A1, A2, A3, B3, and B4 in the exterior part and to zones C1, D1, E1, E2, and F1 in the interior. In overall terms, the mean square error in height ranges from 0.04 m to 0.21 m (Table 3 and Figure 8). 

Table 4 shows a comparison between the data obtained with points located at a distance of up to 2.5 and 7.5 m from the backpack MMS track. The correlation coefficient R^2^ allows the incidence of isolated errors to be analysed when comparing both DSMs. 

## 4. Discussion

The methods for acquiring and processing the data provided by UAV and backpack MMS are of sufficient quality to generate valid DSMs in the study area or similar.

The first part of the work develops the methods of data capture using photogrammetry and a laser scanner with UAV and backpack MMS. The most challenging task was to select the points that could be identified using the different methods. Additionally, 20% of the control points were eliminated due to of the fact that identifying these points in all the methods is not possible.

Except in the case of measurement by photogrammetry, where backpack MMS provides the best precision results with a little under 5 cm in height, the rest of the methods had a precision of around 5 and 10 cm. Better overall results are obtained with UAV methods than with backpack MMS, but this depends on the distance of the points from the axis of the backpack MMS track. Backpack MMS is also better over a short distance under 3 m, with 6.8 cm. At higher distances, the UAV methods obtain a lower precision of over 8 or 9 cm.

In addition to precision, performance and production were also analysed. Manual photogrammetric methods were discarded since they required longer execution times, which represent a higher production cost. The registration of the dense cloud points with UAV proved to be the fastest and most economical method, whereas the registration via MMS gave the best performance, but had a higher production cost.

**Figure 9:**
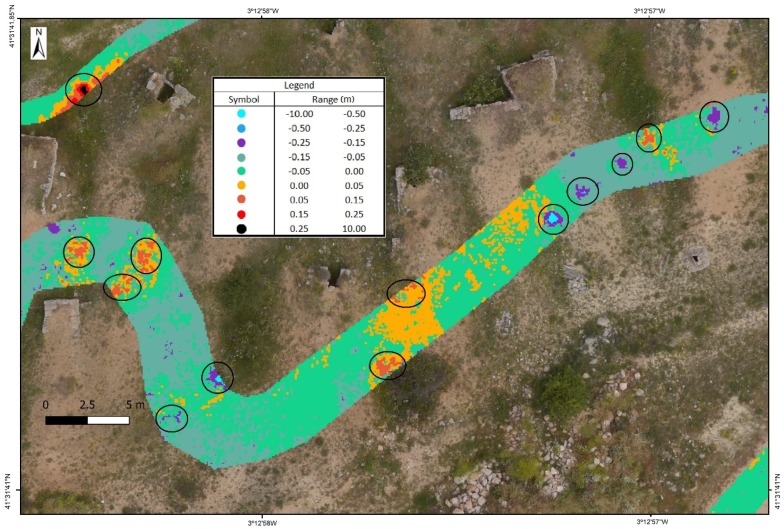
Identification of zones with significant differences between UAV and MMS DSMs.

Given these results, the comprehensive comparison of UAV and backpack MMS point clouds in different zones that are more or less devoid of vegetation and small obstacles allows the analysis of their precision and the influence of each method on the definition of the DSM to be characterised. The precision in clear zones or zones near the track trajectory is 5 cm (Figure 9).

**Figure 10:**
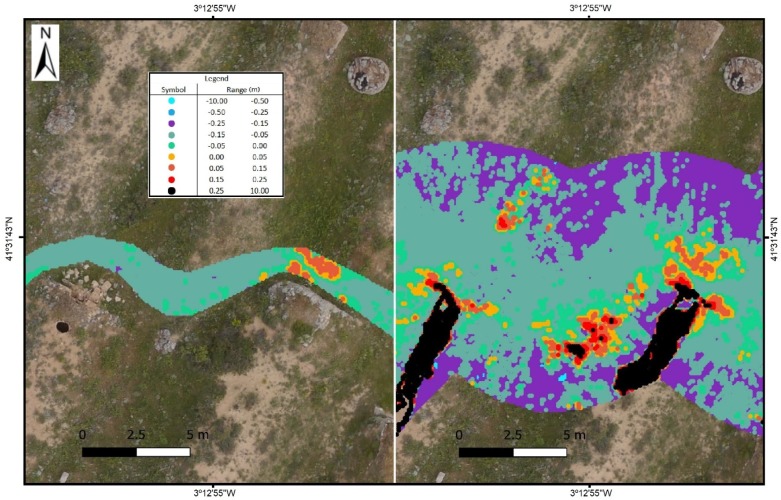
Differences in height according to the type of system, the width of the scan, and the obstacles or concealed elements. (**Left**): example of a zone with a 2.5 m track width. (**Right**): same area with a 15 m track width. Black areas depict concealed zones not registered in the backpack MMS.

More substantial differences are found at greater distances, and measurement problems also arise due to the lack of information or measurements in an orography such as the one in the study area, with holes/gaps or even obstacles, as can be seen at the most extreme points of the model (Figure 10). Although they are few and more distant from the backpack trajectory, they present a proportionally higher error, which can be seen in Table 3 and Figure 7 and Figure 8.

**Figure 11:**
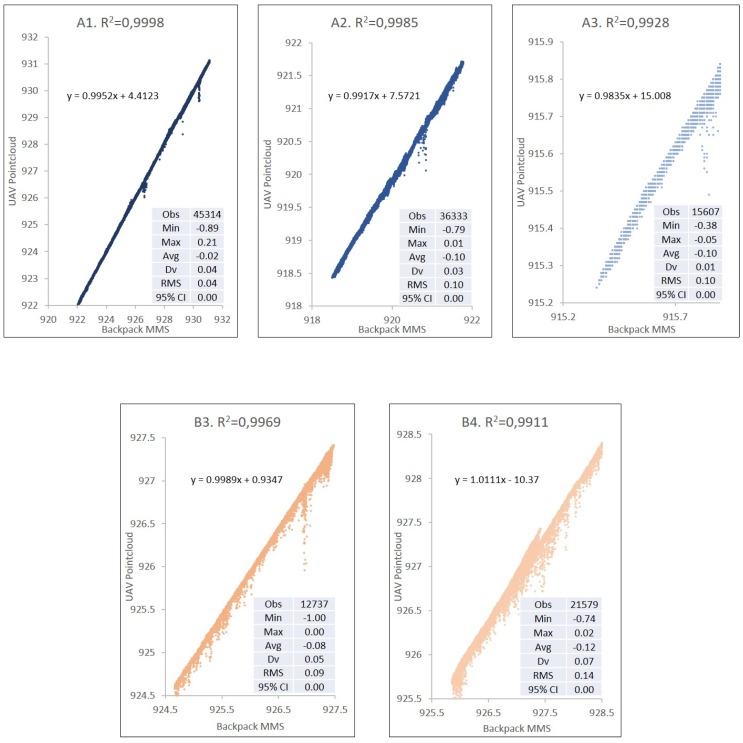
Comparison of UAV vs. backpack MMS by external tracks (**A**,**B**).

**Figure 12:**
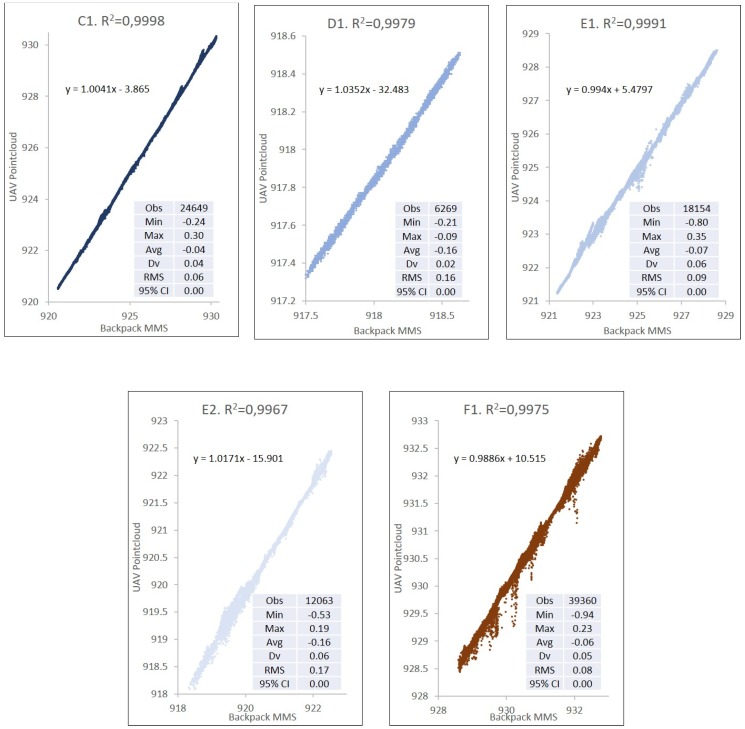
Comparison of UAV vs. backpack MMS by internal tracks (**C**–**F**).

The clearest example of these differences can be seen in the correlation graphs in Figure 11 (external tracks) and Figure 12 (internal tracks), where the different backpack MMS trajectories and their comparison with the UAV points not only show the R^2^ coefficients and the correlation equations indicated above, but also highlight the difference in the number and height of the points outside the trend line for the analysis of each track.

## 5. Conclusions

When comparing the data extracted individually, a similar precision is obtained with an average of around 5 cm in height with both methods, and an average of 10 cm with distance or identification problems. This precision is perfectly acceptable for defining DSMs in this work environment.

Since the precision of both systems is known to be similar in small environments or at short distances from backpack MMS, denser trajectories must be used to reduce errors due to obstacles. A mixed method such as the proposed MMS–UAV technique represents a useful tool for identifying areas that are difficult to determine in the DSM and which lead to the most significant differences in height in the comparison. Given the very high number of points obtained with these procedures, the detection and elimination of these points would make it possible to obtain a DSM with greater precision and a greater level of detail.

The assessment of the DSMs reveals that the tracks followed by backpack mobile mapping present a scarcity of information in some spaces. There are various obstacles and hidden areas in this terrain. In summary, the UAV provides a more homogeneous or stable DSM even though the DSM obtained with backpack mobile mapping is more accurate. This is due to the optimum distance range and the spaces where there are no obstacles or strong rupture lines. Both techniques can, therefore, be considered complementary and reliable for obtaining DSMs for the area where these underground cellars are located. This type of application can help detect deformations in the ground a posteriori.

The use of methods of mass capture offers an excellent opportunity in such a complex area as the exterior surface of the underground cellars of El Plantío in Atauta. These methods have different limitations, such as the irregularity of the terrain, difficult-to-isolate low-growing vegetation, and mobile or fixed obstacles. However, the general precision is high and in line with the data results necessary for their study and preservation.

A novel development is the inclusion of parameters such as the distance to the scanning point, the angles of incidence with regard to the ground, and the study of irregularities in the terrain. A clear comparison of both technologies that conclusively reveals the pros and cons of their use would be impossible without considering these aspects. Their combined use has also been proposed, which may be a source of further improvements in future studies.

The results obtained point to the conclusion that both techniques—albeit not without difficulty—provide DSMs that are capable of defining terrain stability. Due to the registration speed and the precision achieved, both systems allow the assessment of the underground wine cellars. Their use over time will make it possible to establish the necessary priorities to guarantee the conservation of such unique and important spaces such as this site of El Plantío in Atauta.

## Figures and Tables

**Table 1:** UAV and MMS system specifications.

Main Features	Mavinci UAV	Pegasus Backpack
Technology	Lumix-G1 16 Mpx camera	Velodyne VLP16 laser scanner
Measurement technology	Computation from images	Polar distance measurement
Distance measurement	90 m	5–100 m
System resolution	GSD 2.16 cm	Dist. acc. 3 cm at 100 m
DSM resolution	600 points/m^2^	36,000 points/m^2^

**Table 2:** Precision comparison by method and distance to the backpack MMS track (in metres).

Product	Coordinate	0–2		2–3.5		3.5–10	
		Mean	Dev	Mean	Dev	Mean	Dev
Backpack MMS photogrammetry	Horizontal	0.089	0.051	0.049	0.035	0.161	0.109
	Vertical	0.031	0.032	0.025	0.024	0.051	0.033
Backpack MMS point cloud	Horizontal	0.066	0.057	0.032	0.020	0.054	0.027
	Vertical	0.068	0.114	0.090	0.107	0.056	0.049
UAV photogrammetry	Horizontal	0.053	0.034	0.040	0.018	0.047	0.023
	Vertical	0.073	0.053	0.102	0.079	0.096	0.057
UAV point cloud	Horizontal	0.067	0.051	0.033	0.020	0.047	0.022
	Vertical	0.089	0.077	0.118	0.088	0.116	0.063

**Table 3:** Results of the comparison between UAV and backpack MMS DSMs with points measured at a distance of at least 2.5 m from the backpack MMS track.

Track	Perimeter (m)	Area (m^2^)	Distance (m)	UAV Resolution	Backpack MMS Resolution	Grid Resolution	Precision (m)	RMS (m)
A1	370	564	184	357,165	13,694,022	55,578	−0.02	0.04
A2	189	420	90	255,611	11,128,095	41,606	−0.1	0.04
A3	135	196	63	116,012	4,222,443	19,171	−0.1	0.01
A4	95	117	44	69,940	5,290,734	11,562	−0.09	0.03
A5	59	90	26	52,414	7,885,791	8869	−0.16	0.02
B1	98	138	45	80,114	2,921,953	13,628	−0.16	0.02
B2	157	182	77	111,226	5,390,511	17,678	−0.1	0.05
B3	106	159	50	94,737	4,219,886	15,508	−0.08	0.06
B4	236	286	116	180,494	13,471,066	27,982	−0.12	0.07
C1	339	348	168	226,162	9,852,318	33,758	−0.04	0.04
C2	262	339	126	243,346	16,488,277	33,363	−0.09	0.08
D1	69	83	31	52,109	1,691,763	8062	−0.15	0.02
E1	169	232	83	167,189	10,230,845	22,552	−0.07	0.07
E2	109	153	53	105,739	4,740,500	14,961	−0.16	0.07
F1	236	464	116	302,089	14,207,240	45,617	−0.06	0.06

**Table 4:** Comparison of precision when the width is increased from 2.5 to 7.5 m.

	Grid Points		Precision		RMS		R^2^	
Track	5 m	15 m	5 m	15 m	5 m	15 m	5 m	15 m
A1	55,578	233,444	−0.02	−0.07	0.04	0.14	0.9998	0.9959
A2	41,606	135,901	−0.10	−0.19	0.04	0.28	0.9985	0.8108
A3	19,171	88,832	−0.10	−0.1	0.01	0.15	0.9928	0.9806
B3	15,508	71,411	−0.08	−0.1	0.06	0.11	0.9969	0.9792
B4	27,982	144,769	−0.12	−0.12	0.07	0.24	0.9911	0.9968
C1	33,758	197,137	−0.04	−0.09	0.04	0.73	0.9998	0.9598
D1	8062	46,599	−0.15	−0.16	0.02	0.33	0.9979	0.8295
E1	22,552	103,978	−0.07	−0.14	0.07	0.36	0.9991	0.9848
E2	14,961	72,501	−0.16	−0.2	0.07	0.17	0.9967	0.9567
F1	45,617	158,948	−0.06	−0.09	0.06	0.11	0.9975	0.9908
